# Influence of dicarboxylic acid polymer in enhancing the growth and productivity of sweet potato (*Ipomoea batatas* L.) in acidic soil

**DOI:** 10.7717/peerj.14803

**Published:** 2023-02-02

**Authors:** Le Van Dang, Ngo Ngoc Hung, Le Phuoc Toan, Ngo Phuong Ngoc

**Affiliations:** College of Agriculture, Can-Tho University, Can Tho, Vietnam

**Keywords:** Acid soils, AVAIL, Available phosphorus, Mekong Delta, Nutrient uptake, Root crops

## Abstract

The available phosphorus (P) in acid sulfate soils (ASSs) is low because of fixation by aluminum (Al) and iron (Fe), resulting in decreased P use efficiency and crop yield. At present, the use of dicarboxylic acid polymer (DCAP) coated on P fertilizer is expected to improve P use efficiency and plant productivity. However, the influence of DCAP on P solubility and on the yield of sweet potato cultivated in acidic soils has not been elucidated. Thus, the aimed of this study was to evaluate the effect of the use of DCAP-coated P fertilizer on the availability and nutrient uptake of P as well as the yield of sweet potato. Under the greenhouse condition, the use of DCAP significantly improved P availability (~3 mg P kg^−1^), increasing tuber diameter and length by ~0.5 and ~1.0 cm, respectively. Thus, the productivity of sweet potato in the treatment 40-kg P_2_O_5_ and 60-kg P_2_O_5_ ha^−1^ coated with DCAP was higher by about 100 g pot^−1^ than that in the same rate of P fertilizers (40- and 60-kg P_2_O_5_ ha^−1^) not coated with DCAP. In the field experiment, P accumulation (82.7 kg P_2_O_5_ ha^−1^) and tuber yield (22.0 t ha^−1^) in the treatment of DCAP-coated with 60-kg P_2_O_5_ ha^−1^ were not significantly different compared with that in the treatment of 80-kg P_2_O_5_ ha^−1^ (82.1 kg P_2_O_5_ and 21.7 t ha^−1^, respectively). Furthermore, the use of DCAP combined with 75% P fertilizer increased the P availability by the same amount as that with the use of 100% P fertilizer. Hence, the use of DCAP reduced about 25% of the chemical P fertilizer applied in soil.

## Introduction

In the Vietnamese Mekong Delta (VMD), acid sulfate soils (ASSs) occupy an area of about 1.65 million ha. The VMD is divided into four principal areas: the (i) Ca Mau Peninsula, (ii) Long Xuyen Quadrangle, (iii) Plain of Reeds, and (iv) Depressed of Hau River (Xuan & Matsui, 1998). According to Alwi, Yusuf & Fahmi (2021), ASSs face several obstacles, including: soil acidity, the high concentration of Al^3+^ and Fe^2+^, and the low availability of phosphorus (P) and potassium (K) nutrients. The acidity of ASSs is affected by a number of factors, including the presence of pyrite, iron oxide-hydroxide minerals, sulfates, organic matter, and neutralizing agents and the hydrological conditions of the land (Alwi, Yusuf & Fahmi, 2021). Furthermore, soil acidification causes the loss of base cations, an increase in aluminum (Al) saturation and a decrease in plant productivity; consequently, severe acidification can cause nonreversible clay mineral dissolution and a reduction in cation exchange capacity, accompanied by structural deterioration (Goulding, 2016).

Rice is considered the main crop of the VMD region. However, the economics of rice cultivation is not so lucrative (Dang, Hung & Phuong, 2018). Various studies have indicated that root crops (yam, sweet potato, and cassava) have the ability to grow and have high economic efficiency in the acidic soils of VMD (Quyen, Dang & Phuong, 2016; Dang et al., 2016a, 2016b). Therefore, the Vietnamese government decided to replace rice with upland crops (sweet potato, yam, cassava, *etc*.). Sweet potato (*Ipomoea batatas* L.) is an important food widely cultivated in tropical countries (Escobar-Puentes et al., 2022). It is a rich source of vitamin A, vitamin C, manganese, copper, digestive fiber, vitamin B6, potassium (K), and iron (Fe) (Neela & Fanta, 2019). The stem and tuber of sweet potato are consumed as food by humans and livestock (Chandrasekara & Kumar, 2016; Akoetey, Britain & Morawicki, 2017). Sweet potato is one of the three main root crops in the VMD (Lang et al., 2020). In 2020, the total productivity of sweet potato in Vietnam was 300,000 tons (GSO, 2021). Most of the sweet potato yield is cultivated in ASSs, with an area of about 15,000 ha (Lang et al., 2020). In the VMD, Dong Thap and Vinh Long are the two provinces with the highest total production of sweet potato (Lang et al., 2020). Thus, these areas are considered to be of high value in the region for both domestic markets and exports (Dang et al., 2016a). According to Hien et al. (2015), the profit from sweet potato was higher by about five-folds as compared with that of rice. Thus, the sweet potato industry could be an important means to to reduce poverty and improve farmer’s livelihood. Phosphorus (P) is a vital element for the growth and development of sweet potato (Cruz et al., 2016). It plays a significant role in the metabolic process and tuber development (Kareem et al., 2020). In addition, P is essential in respiration, photosynthesis, and in the formation of pyrophosphate bonds that allow energy transfer (Cruz et al., 2016). However, the P use efficiency on acidic soils is limited given the interaction of P with Fe and Al, producing different phosphate compounds that reduce P availability in soil (Hopkins, Horneck & MacGuidwin, 2014; Ikhajiagbe et al., 2020; Johan et al., 2021). Therefore, about 70% of P is not used after application in soil (Bhattacharya, 2019). According to Alori, Glick & Babalola (2017), P is an important element that affects the growth and productivity of plants. Hence, to reduce P deficiency in acidic soils, farmers have tended to overuse chemical P fertilizers, which may lead to an increase in the cost of production with reduced profits (Noor et al., 2017). A previous study has reported that P fertilizer application significantly improved P accumulation and dry matter production of purple-fleshed sweet potato, resulting in an increased tuber yield (Liu et al., 2022). Another study indicated that the supply of P fertilizer greatly enhanced the yield of sweet potatos (Mugai, Mworia & Martin, 2022).

Many studies have reported that using dicarboxylic acid polymer (DCAP)-coated P fertilizer positively affected the growth and yield of crops (Jeffrey et al., 2017; Hopkins et al., 2018). It has been reported that DCAP supplies a compound with high negative-charge density (Jeffrey & Hopkins, 2013). When DCAP was applied in the soil, it prevented cations Fe and Al from precipitating P, hence, enhancing the solubility of P, resulting in increased P uptake (Hopkins, 2015). However, each soil group and each crop species have different yields and growth responses to DCAP. The hypothesis underlying this work is that in acidic soils, the use of DCAP may lead to improved crop yield due to an increase in the available nutrient (P) in the soil. This study aimed to determine (i) whether the use of DCAP in ASSs enhances the P availability in soil and (ii) the yield of sweet potato.

## Materials and Methods

### Study sites, climate, and soils

The soil used for the greenhouse experiment was obtained from the Plain of Reeds and Depressed of the Hau River. These are located in the Dong Thap Province (10°11′23.6″ N; 105°47′47.9″ E) and Vinh Long (10°09′49.2″ N 105°45′47.7″ E), respectively. Field experiments were approved by Tran Van Bay and Nguyen Van Thuan. They are farmers and own these soils, with the contract number: 101/2019/HĐ-NCKH and 105/2019/HĐ-NCKH, respectively.

The VMD has a tropical climate and two seasons, namely, the dry season, which occurs from December to April, and the wet season, which occurs between May and November. The current work was conducted during the dry season in 2018 (greenhouse experiment) and 2019 (field experiment). The average temperature in the months did not vary enough, the average temperature of the tank during the experiment was 28 °C, and the total sunshine hours in the month was 240.

Field trials were conducted at the same locations where soils were collected for the pot studies. Before conducting the pot experiments, the physicochemical properties of the soil were investigated at two depths (0–15 and 15–30 cm). The results of the investigation of the chemical and physical characteristics of the soil in the study sites are presented in Table 1. The soils in the study sites were classified as orthi–thionic–fluvisols according to World Reference Base for Soil Resources (WRB) (2015).

**Table 1:** Initial soil characteristics in two study locations.

a. Soil chemical properties												
Site	Depth (cm)	pH_H2O_ (1:2.5)	EC (mS cm^−1^)	CEC (cmol_c_ kg^−1^)	Exchangeable cations (cmol_c_ kg^−1^)				AP (mg P kg^−1^)	TP (% P)	SOM (%)	TN (%)
					Na^+^	K^+^	Ca^2+^	Mg^2+^				
Dong Thap	0–15	4.12	0.82	19.2	0.31	0.21	3.18	2.37	19.2	0.08	4.21	0.13
	15–30	4.23	1.12	18.9	0.42	0.16	4.22	2.95	16.8	0.05	3.56	0.09
Vinh Long	0–15	3.96	0.75	20.1	0.56	0.19	3.56	2.38	15.9	0.06	4.59	0.11
	15–30	4.10	0.66	19.6	0.38	0.32	3.29	3.26	16.7	0.09	4.10	0.10
**b. Soil physical and toxicity parameters**												
Site	Depth (cm)	BD (g cm^−3^)	Total porosity (%)	AWC (mm m^−3^)	Soil particles (%)			Fe^2+^ (mg kg^−1^)	Fe_2_O_3_ (%)	Sulfate S (μg S g soil^−1^)	H^+^ (cmol_c_ kg^−1^)	Al^3+^ (cmol_c_ kg^−1^)
					Sand	Silt	Clay					
Dong Thap	0–15	1.12	54.2	254	3.20	43.5	53.3	42.6	0.42	12.1	5.93	0.33
	15–30	1.08	55.6	236	2.50	47.5	50.0	38.9	0.35	7.82	4.62	0.26
Vinh Long	0–15	1.05	54.1	249	2.90	42.9	54.2	47.1	0.51	9.37	5.56	0.39
	15–30	1.10	52.9	250	2.40	41.9	55.7	40.8	0.39	10.4	5.05	0.27

**Note:**

EC, electrical conductivity; CEC, cation exchange capacity; AP, available phosphorus; TP, total phosphorus; TN, total nitrogen; BD, soil bulk density; AWC, available water capacity.

### DCAP preparation and sweet potato variety

The DCAP used in the present work is a commercial product of Verdesian Life Sciences, USA. Its commercial name is “AVAIL^®^ for Granular Phosphate Fertilizers”.

The sweet potato variety “Japanese purple” was used for this study because it is popularly cultivated in the VMD, and it has a higher economy compared with that of local sweet potatos (Dang et al., 2016a). The “Japanese purple” variety is large, purple in color, and has fat stems and little branching (Lang et al., 2020). Furthermore, the “Japanese purple” variety has vigorous growth ability, especially in ASSs, with a growth duration of 105–120 d and a productivity of about 20–25 tons ha^−1^ (Hien et al., 2015). Its tubers are oblong, with smooth skin and dark yellow flesh; moreover, its dry matter content is 27–33%. This variety is suitable for fresh eating, processing, and exporting (Lang et al., 2020).

### Experimental layout

Pot experiments in two different ASSs were conducted in a greenhouse at the Soil Science Department, College of Agriculture, Can Tho University, Can Tho City, Vietnam (10°01′44.8″ N, 105°46′00.5″ E). The trials were laid out according to a completely randomized design (CRD) with four replicates, with each replicate including a pot. The total number of pots in the current study was 48. Each soil group had 24 pots. The treatments included: (i) 40-kg P_2_O_5_ ha^−1^; (ii) 40-kg P_2_O_5_ ha^−1^ coated with DCAP; (iii) 60-kg P_2_O_5_ ha^−1^; (iv) 60-kg P_2_O_5_ ha^−1^ coated with DCAP; (v) 80-kg P_2_O_5_ ha^−1^; and (vi) 80-kg P_2_O_5_ ha^−1^ coated with DCAP.

After collecting the soils from the two aforementioned locations, sweet potato residues (leaves, stem, and roots) were eliminated during soil mixing and drying in air. The pots were 60 cm in diameter and 40 cm in depth. Each pot was filled with 30 kg of soil with a humidity of 7–8%. Two sweet potato cuttings that were 30-cm long were planted in each pot.

The field experiment was carried out in a randomized complete block design at the two ASS sites, which are described in ‘Study sites and soils’. The treatments were as follows: (i) 100% of the recommended dose of P fertilizer (80-kg P_2_O_5_ ha^−1^); (ii) 100% of the recommended dose of P fertilizer (80-kg P_2_O_5_ ha^−1^) coated with DCAP; (iii) 75% of the recommended dose of P fertilizer (60-kg P_2_O_5_ ha^−1^); and (iv) 75% of the recommended dose of P fertilizer (60-kg P_2_O_5_ ha^−1^) coated with DCAP. The experiment was conducted with four replicates at each study site.

Before the start of the experiment, the field was cleared of weeds, and raised beds (80-cm wide, 50-cm high) were created, with a distance of 35 cm between the raised beds. Each plot size was 4 m^2^ (5 m long × 0.8 m wide). The Japanese purple cuttings were 30-cm long, cultivating two rows of cuttings on each raised bed, with two thirds of the cuttings buried in the ground, and a density of 200,000 cuttings ha^−1^. Constant watering was required to make sure that the soil was moist enough. Sweet potato was harvested when the stem growth slowed down, the leaves turned yellow and started falling, the tuber became smooth, and few extra roots were observed (4 months after planting).

### Fertilization formula and timing

The application rates of N, P_2_O_5_, and K_2_O (100, 80, and 200 kg ha^−1^, respectively) for the Japanese purple were in accordance with the recommendations by Hien et al. (2015). The NPK fertilizers used in this study include urea (46% N), diammonium phosphate (18% N–46% P_2_O_5_), and K oxide (60% K_2_O).

The timing and rates for NPK fertilizer application for the sweet potato in the VMD was based on the study by Hien et al. (2015). At 5 days after planting (DAP), 15% of the total N and 40% of the total P were added; at 15 DAP, 15% and 40% of the total N and P were added, respectively; and at 25 DAP, 40%, 20%, and 20% of the total N, P, and K were added, respectively. At 45 DAP, 15% of the total N and 40% of the total K were added, and at 65 DAP, N and K were added at the rate of 15% and 40%, respectively.

### Soil and plant analysis

Soil sample collection was performed during the harvest of sweet potato. In both the pot and field trials, using a soil auger (diameter of about 3 cm) was used to take five soil cores at a depth of 0–15 cm (topsoil) and 15–30 cm (subsoil) following a zigzag pattern. After that, the collected soil cores in each layer were mixed into one composite sample of about 450 g. For the analysis, the soil samples were air-dried for 14 days, crushed, and sieved through 0.5- and 2.0-mm meshes. The soil and plant analysis methods are presented in Table 2.

**Table 2:** Soil and plant sampling analysis procedures.

Parameters	Method employed	Reference
*I. soil*		
Soil acidity (pH)	pH meter	Houba, Vanderlee & Novozamsky (1995)
Al^3+^	Titrimetric method	Abreu-Junior, Muraoka & Lavorante (2003)
Fe^2+^	Ferrozine method	Viollier et al. (2000)
Available P	Bray 2	Bray & Kurtz (1945)
Total P	Colorimetric orthophosphate	Sommers & Nelson (1972)
*I. plant*		
Total P	Colorimetric P	Adesanwo et al. (2013)

In the pot trial, at 120 DAP, two tubers and a stem of sweet potato in each treatment was selected for biomass determination and P accumulation. The tubers and stem were washed with tap water to eliminate the soil, and the root was removed; then, they were weighed, minced, and dried at 68 °C in an oven until constant mass was reached. Finally, they were weighed again, and the values were converted to g pot^−1^. In the field study, at 120 DAP, six tubers and three stems were washed and weighed and then minced and dried at 68 °C in an oven until constant weight was reached; the values were converted to kg ha^−1^.

P nutrient uptake was calculated using the P concentration in each part multiplied by the dry weight of that part. The total uptake of P was equal to the sum of the uptake of that nutrient in the tubers and stems.

### Yield components and productivity

In each pot, all tubers were harvested at 120 DAP and then washed, counted, measured, and weighed to determine the number of tuber pot^−1^, tuber diameter (cm) and length (cm), and tuber yield (g pot^−1^), respectively.

For the field trial, tuber yield (kg m^2^) was determined by harvesting all the tubers in each plot, and then the values were converted to tons ha^−1^.

### Statistical analysis

In the present work, SPSS software (version 20.0; SPSS Inc., Chicago, IL, USA) was used for statistical analysis. Analysis of variance was employed to compare the differences between the means among treatments using Duncan’s *post hoc* test at *p* < 0.05, 0.01, and 0.001. The relationship between tuber yield and the P concentration was determined using Pearson’s correlation coefficient.

## Results

### Influence of DCAP on available P and yield of sweet potato under the greenhouse condition

#### Soil chemical properties

Soil acidity in two depths in greenhouse conditions was not affected by the treatments and sites (Table 3). In the soil in Dong Thap, the pH values in the topsoil (0–15 cm) and subsoil (15–30 cm) ranged from 3.98 to 4.13 and from 4.31 to 4.41, respectively. Contrarily, in the soil in Vinh Long, the pH values in the topsoil and subsoil ranged from 4.03 to 4.12 and 4.08 to 4.20, respectively. The concentration of soil available P was significantly higher in the DCAP-coated P fertilizer treatments than that without DCAP in both study locations. The P availability in the treatment of 80-kg P_2_O_5_ ha^−1^ coated with DCAP was the highest in the two study sites. The total P in soil was not affected by the treatments and sites. Similarly, the concentrations of Fe and Al were not different among the treatments.

**Table 3:** Effects of DCAP-coated P fertilizer on soil quality.

Location	Depth (cm)	Treatment	pH_H2O_ (1:2.5)	P availability (mg P kg^−1^)	Total P (% P)	Fe^2+^ (mg kg^−1^)	Al^3+^ (cmol_c_ kg^−1^)
Dong Thap	0–15	T1	4.08	18.2^f^	0.07	41.5	0.31
		T2	4.01	22.2^e^	0.08	40.3	0.32
		T3	4.13	26.0^d^	0.09	40.7	0.32
		T4	3.98	28.6^c^	0.08	42.9	0.36
		T5	4.11	30.0^b^	0.09	42.2	0.35
		T6	4.10	32.9^a^	0.09	42.6	0.27
		*P-value*	ns	***	ns	ns	ns
	15–30	T1	4.31	17.5^f^	0.09	37.1	0.30
		T2	4.27	21.6^e^	0.09	37.1	0.30
		T3	4.27	24.6^d^	0.08	36.5	0.28
		T4	4.41	27.1^c^	0.08	36.9	0.31
		T5	4.39	29.3^b^	0.07	37.7	0.26
		T6	4.29	31.8^a^	0.09	37.5	0.26
		*P-value*	ns	***	ns	ns	ns
Vinh Long	0–15	T1	4.06	15.7^d^	0.08	44.4	0.42
		T2	4.08	19.0^c^	0.09	45.4	0.43
		T3	4.03	21.0^c^	0.08	44.8	0.45
		T4	4.06	27.6^b^	0.09	45.1	0.41
		T5	4.06	31.7^a^	0.08	46.4	0.48
		T6	4.12	32.8^a^	0.10	45.8	0.41
		*P-value*	ns	***	ns	ns	ns
	15–30	T1	4.17	17.9^d^	0.09	42.6	0.28
		T2	4.10	22.8^c^	0.10	40.2	0.29
		T3	4.20	25.2^c^	0.09	41.7	0.30
		T4	4.15	30.6^b^	0.11	43.2	0.28
		T5	4.11	35.0^a^	0.09	42.6	0.29
		T6	4.08	34.5^a^	0.10	42.9	0.30
		*P-value*	ns	***	ns	ns	ns

**Note:**

T1, 40-kg P_2_O_5_ ha^−1^; T2, 40-kg P_2_O_5_ ha^−1^ coated with DCAP; T3, 60-kg P_2_O_5_ ha^−1^; T4, 60-kg P_2_O_5_ ha^−1^ coated with DCAP; T5, 80-kg P_2_O_5_ ha^−1^; T6, 80-kg P_2_O_5_ ha^−1^ coated with DCAP. The different letters in each column indicate significant differences at *p* < 0.001 (***) according to Duncan’s multiple range test; ns, not significant.

### The components of sweet potato yield and productivity

The yield components (excluding the number of tubers) and productivity of sweet potato were significantly higher in the DCAP-coated treatments than those not coated with DCAP in both ASSs from Dong Thap and Vinh Long (Table 4). The results indicated that the yield components and productivity of sweet potato in the treatment of 60-kg P_2_O_5_ ha^−1^ coated with DCAP were not significantly different compared with those in the treatment of 80-kg P_2_O_5_ ha^−1^. In the Dong Thap soil, the harvest index (HI) was significantly improved by the treatments. For example, HI increased by 0.05, 0.06, and 0.06 in the treatments of 80-kg P_2_O_5_ ha^−1^ coated with DCAP, 80-kg P_2_O_5_ ha^−1^, and 60-kg P_2_O_5_ ha^−1^ coated with DCAP, respectively, as compared to that in the treatment of 40-kg P_2_O_5_ ha^−1^. In the Vinh Long soil, the DCAP-coated P fertilizer did not affect the HI of sweet potato, except for the treatment 40-kg P_2_O_5_ ha^−1^ coated with DCAP. The use of DCAP-coated on the P fertilizer at a dose of 40 kg P_2_O_5_ ha^−1^ was higher than that in the same dose of P fertilizer but without coated DCAP.

**Table 4:** Impact of DCAP-coated P fertilizer on yield components and tuber productivity.

Location	Treatment	Number of tuber pot^−1^	Tuber diameter (cm)	Tuber length (cm)	Yield (kg pot^−1^)	HI
Dong Thap	T1	5.68	3.35^c^	15.6^d^	0.59^c^	0.67^c^
	T2	5.59	3.94^b^	17.0^cd^	0.72^b^	0.70^b^
	T3	5.63	3.65^bc^	18.7^bc^	0.79^b^	0.71^ab^
	T4	5.60	4.73^a^	19.9^ab^	0.90^a^	0.73^a^
	T5	5.45	4.75^a^	20.8^a^	0.90^a^	0.73^a^
	T6	5.63	4.91^a^	20.6^a^	0.87^a^	0.72^a^
	*P-value*	ns	***	***	***	***
Vinh Long	T1	5.55	3.34^c^	14.1^c^	0.56^c^	0.65^b^
	T2	5.53	3.80^b^	15.7^b^	0.72^b^	0.69^a^
	T3	5.48	3.85^b^	17.1^b^	0.80^ab^	0.71^a^
	T4	5.65	4.44^a^	20.1^a^	0.84^a^	0.71^a^
	T5	5.55	4.38^a^	20.3^a^	0.87^a^	0.72^a^
	T6	5.68	4.76^a^	20.7^a^	0.88^a^	0.72^a^
	*P-value*	ns	***	***	***	**

**Note:**

The different letters in each column indicate significant differences at *p* < 0.001 (***) according to Duncan’s multiple range test; ns, not significant; T1, 40-kg P_2_O_5_ ha^−1^; T2, 40-kg P_2_O_5_ ha^−1^ coated with DCAP; T3, 60-kg P_2_O_5_ ha^−1^; T4, 60-kg P_2_O_5_ ha^−1^ coated with DCAP; T5, 80-kg P_2_O_5_ ha^−1^; T6, 80-kg P_2_O_5_ ha^−1^ coated with DCAP; HI, harvest index.

### P uptake

Table 5 demonstrates that the concentrations of P in the stem and tuber were significantly increased by DCAP. In particular, the values of the P content in the stem and tuber ranged from 0.63% to 0.70% and 0.42% to 0.54% in the Dong Thap soil and from 0.71% to 0.76% and 0.44% to 0.54 % in the Vinh Long soil, respectively. The DCAP-coated P fertilizer increased the dry tuber and stem biomass of the sweet potato compared with that of the same dose of P not coated with DCAP. Likewise, significant differences were observed at *p* < 0.01 among treatments affected by P fertilizers and DCAP in dry biomass (stem and tuber values). DCAP-coated P fertilizers had a higher biomass than those not coated with DCAP. The total P uptake was significantly improved by the treatments in the Dong Thap and Vinh Long soils.

**Table 5:** Effects of DCAP-coated P fertilizer on P uptake.

Location	Treatment	P content (% P_2_O_5_)		Dry biomass (g pot^−1^)		Total P uptake (g P_2_O_5_ pot^−1^)
		Stem	Tuber	Stem	Tuber	
Dong Thap	T1	0.58^c^	0.43^c^	145^d^	293^c^	209^c^
	T2	0.65^b^	0.47^b^	157^c^	361^b^	273^b^
	T3	0.65^b^	0.46^bc^	158^bc^	393^b^	285^b^
	T4	0.72^a^	0.53^a^	166^a^	450^a^	357^a^
	T5	0.73^a^	0.54^a^	164^ab^	450^a^	360^a^
	T6	0.72^a^	0.55^a^	167^a^	435^a^	360^a^
	*P-value*	***	***	***	***	***
Vinh Long	T1	0.60^d^	0.44^c^	148^c^	281^c^	212^c^
	T2	0.66^c^	0.51^b^	160^b^	361^b^	287^b^
	T3	0.68^bc^	0.52^b^	161^b^	400^ab^	318^ab^
	T4	0.70^ab^	0.57^a^	170^a^	420^a^	359^a^
	T5	0.72^a^	0.59^a^	172^a^	435^a^	378^a^
	T6	0.72^a^	0.58^a^	173^a^	440^a^	380^a^
	*P-value*	***	***	***	***	***

**Note:**

The different letters in each column indicate significant differences at *p* < 0.001 (***) according to Duncan’s multiple range test; ns, not significant; T1, 40-kg P_2_O_5_ ha^−1^; T2, 40-kg P_2_O_5_ ha^−1^ coated with DCAP; T3, 60-kg P_2_O_5_ ha^−1^; T4, 60-kg P_2_O_5_ ha^−1^ coated with DCAP; T5, 80-kg P_2_O_5_ ha^−1^; T6, 80-kg P_2_O_5_ ha^−1^ coated with DCAP.

### Reducing P fertilizer by using DCAP under field conditions

#### Soil quality properties

Soil pH was not affected by the treatments and the study sites (Table 6). At Dong Thap, the soil pH values varied from 4.32 to 4.50 at a depth of 0–15 cm and from 4.39 to 4.46 at a depth of 15–30 cm. In Vinh Long, these values ranged from 4.16to 4.32 and from 4.06 to 4.30, respectively. The content of available P in soil significantly increased in the treatment of 75% P + DCAP compared with that in the 75% P treatment in both study sites. Contrarily, the total P content in soils from Dong Thap and Vinh Long was not influenced by DCAP. Similar to the total P, the use of DCAP did not affect the concentration of Fe and Al in both study sites.

**Table 6:** Impacts of DCAP-coated P fertilizer on soil chemical in two ASSs.

Location	Depth (cm)	Treatment	pH_H2O_ (1:2.5)	P availability (mg P kg^−1^)	Total P (% P)	Fe^2+^ (mg kg^−1^)	Al^3+^ (cmol_c_ kg^−1^)
Dong Thap	0–15	100% P	4.36	28.9^a^	0.09	32.7	0.26
		100% P + DCAP	4.32	28.1^a^	0.11	33.7	0.24
		75% P	4.49	18.2^b^	0.10	35.0	0.26
		75% P + DCAP	4.50	30.4^a^	0.11	36.4	0.29
		*P-value*	ns	***	ns	ns	ns
	15–30	100% P	4.39	28.7^a^	0.11	37.3	0.29
		100% P + DCAP	4.46	27.9^a^	0.10	34.0	0.26
		75% P	4.46	18.1^b^	0.09	32.2	0.34
		75% P + DCAP	4.43	27.6^a^	0.09	36.1	0.33
		*P-value*	ns	***	ns	ns	ns
Vinh Long	0–15	100% P	4.16	25.2^a^	0.11	44.2	0.37
		100% P + DCAP	4.26	26.1^a^	0.08	44.0	0.40
		75% P	4.32	15.2^b^	0.09	38.5	0.38
		75% P + DCAP	4.28	26.2^a^	0.09	38.6	0.32
		*P-value*		***	ns	ns	ns
	15–30	100% P	4.06	25.9^a^	0.08	41.5	0.27
		100% P + DCAP	4.30	24.2^a^	0.12	40.2	0.31
		75% P	4.23	15.6^b^	0.10	36.5	0.25
		75% P + DCAP	4.23	25.3^a^	0.10	36.4	0.29
		*P-value*	ns	***	ns	ns	ns

**Note:**

The different letters in each column indicate significant differences at *p* < 0.001 (***) according to Duncan’s *post hoc* test. 100% P, 80-kg P_2_O_5_ ha^−1^; 100% P + DCAP, 80-kg P_2_O_5_ ha^−1^ coated with DCAP; 75% P, 60-kg P_2_O_5_ ha^−1^; and 75% P + DCAP, 60-kg P_2_O_5_ ha^−1^ coated with DCAP.

### Sweet potato productivity, dry biomass, and P uptake

The use of DCAP significantly increased the sweet potato yield by comparison with that of the other treatments in the two study sites (Table 7). The sweet potato productivity in the treatments of 75% P + DCAP, 100% P, and 100% P + DCAP was 22.0, 21.7, and 21.5 t ha^−1^, respectively. In Vinh Long, these values were 24.4, 23.9, and 24.1 t ha^−1^. The results in Table 7 indicate that the P concentrations in the stem and tuber are significantly different among treatments. The P concentration in the stem and tuber in 75% P + DCAP was higher than that in 75% P in both study sites. Meanwhile, no significant difference was observed between the treatments of 75% P + DCAP and 100% P. Likewise, the application of 75% P + DCAP improved the dry biomass (stem and tuber) and total P uptake compared with the addition of 75% P without DCAP.

**Table 7:** Influences of DCAP-coated P fertilizer on the yield and P uptake of sweet potato.

Location	Treatment	Tuber yield (t ha^−1^)	P concentration (% P_2_O_5_)		Dry biomass (t ha^−1^)		Total P accumulation (kg P_2_O_5_ ha^−1^)
			Stem	Tuber	Stem	Tuber	
Dong Thap	100% P	21.7^a^	0.71^a^	0.50^a^	5.00^a^	9.42^a^	82.1^a^
	100% P + DCAP	21.5^a^	0.73^a^	0.50^a^	5.05^a^	9.45^a^	83.5^a^
	75% P	19.6^b^	0.65^b^	0.43^b^	4.58^b^	8.67^b^	67.1^b^
	75% P + DCAP	22.0^a^	0.71^a^	0.49^a^	5.07^a^	9.45^a^	82.7^a^
	*P-value*	*	***	*	***	***	***
Vinh Long	100% P	23.9^a^	0.70^a^	0.46^a^	5.18^a^	10.3^a^	83.7^a^
	100% P + DCAP	24.1^a^	0.71^a^	0.47^a^	5.10^a^	10.1^a^	83.5^a^
	75% P	20.6^b^	0.64^b^	0.42^b^	4.66^b^	8.83^b^	66.5^b^
	75% P + DCAP	24.4^a^	0.71^a^	0.46^a^	5.13^a^	10.2^a^	83.5^a^
	*P-value*	***	*	*	***	***	***

**Note:**

The different letters in each column indicate significant differences at *p* < 0.05 (*) and *p* < 0.001 (***) according to Duncan’s *post hoc* test. 100% P, 80-kg P_2_O_5_ ha^−1^; 100% P + DCAP, 80-kg P_2_O_5_ ha^−1^ coated with DCAP; 75% P, 60-kg P_2_O_5_ ha^−1^; and 75% P + DCAP, 60-kg P_2_O_5_ ha^−1^ coated with DCAP.

### The relationship between soil available P and sweet potato yield

#### Greenhouse condition

**Figure 1:**
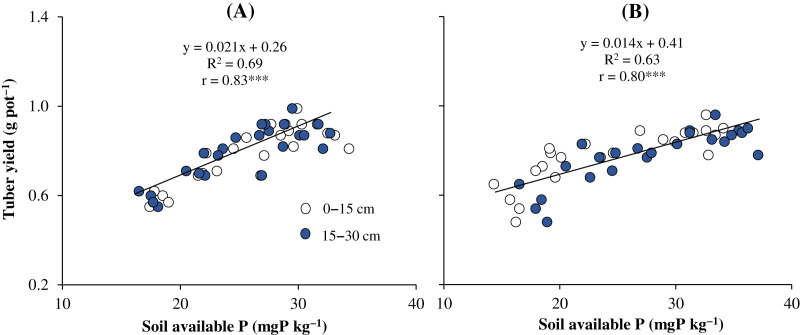
The correlation between sweet potato productivity and soil available P in the Dong Thap soil (A) and Vinh Long soil (B) under the greenhouse condition. Asterisks (***) indicates significant difference at *p* < 0.001.

In this study, available P supply was significantly correlated with the tuber yield of sweet potato (*r* = 0.83***) in the Dong Thap soil (Fig. 1A). The relationship between sweet potato productivity in the Vinh Long soil and P availability was weak (*r* = 0.80***) (Fig. 1B). Figure 1 demonstrates that the soil available P is the key factor affecting sweet potato yield in the pot experiment.

#### Field condition

**Figure 2:**
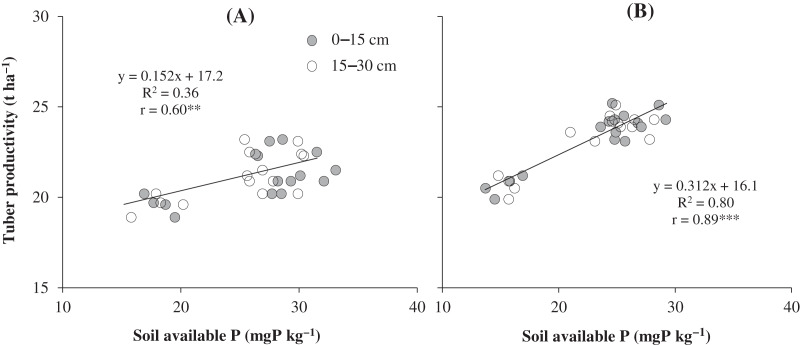
The relationship between sweet potato yield and soil P availability in the two field study sites: (A) Dong Thap and (B) Vinh Long. Asterisks (** and ***) indicate significant difference at *p* < 0.01 and *p* < 0.001, respectively.

There was a strong positive (*r* = 0.60**) relationship between soil P availability and sweet potato productivity in Dong Thap (Fig. 2A). Enhancing the amount of P availability in soil leads to an increase in sweet potato yield. Figure 2B also shows that there is a strong positive and significant correlation between soil available P and sweet potato yield in the Vinh Long soil.

## Discussion

The initial physicochemical characteristics of ASSs from the two study locations showed that they have high soil acidity and toxicity due to Fe and Al concentrations (Table 1), resulting in a decrease in the available P content in soil. According to Margenot et al. (2017), declining P availability in ASSs is due to the immobilized P that was precipitated by Al and Fe ions to create insoluble compounds (AlPO_4_ and FePO_4_). These forms of P are not easy for plant uptake and accumulation (Ngoc et al., 2022). Many previous studies have demonstrated that there is a significant positive correlation between soil available P and sweet potato yield (Kareem et al., 2020; Liu et al., 2022; Mugai, Mworia & Martin, 2022). However, these studies were conducted in soil groups with high soil pH and low Al and Fe concentrations. In the current study, the use of DCAP was aimed at preventing P fixed by Fe or Al, creating insoluble P compounds in soil.

The results of the trial in the greenhouse indicated that the DCAP-coated P fertilizer significantly increased the P availability content compared to the control (Table 3). Enhancement of soil available P led to better yield components (tuber diameter and length) and productivity of sweet potato (Table 4). P is an essential nutrient for the growth and yield of root crops (Kahsay & Moral, 2019) because it plays an important role in the metabolic processes and root development (Kareem et al., 2020). According to Hopkins et al. (2018), DCAP compound significantly improved P solubility in soil solution. Mooso, Tindall & Hettiarachchi (2013) demonstrated that the use of DCAP along with P fertilizers enhanced P availability. Therefore, DCAP is considered a beneficial choice for improving P use efficiency. The dose of P for crops depends on their type and their ability to take up P in soil (Hopkins, 2015). Kareem et al. (2020) observed that the tuber yield of sweet potato significantly increased with the application of P fertilizer. It should be noted that sweet potato is a crop requiring high P concentrations for tuber formation (Lv & Lu, 2021). Thus, an increased available P content in soil increases sweet potato yield (Fig. 1). These results were in agreement with those from the study by Cruz et al. (2016) and Kareem et al. (2020). They reported that the use of P fertilizers improved P availability, leading to increased productivity of sweet potato.

DCAP is a complex fertilizer that contains 18% N and 46% P. These P ions are easily attracted by the heavy metals Fe and Al, thus reducing the available P content in crop soils (Johan et al., 2021). Coating of P fertilizer with DCAP is the best choice for increasing P use efficiency due to the decreased release of P ions in soil solution and for preventing P fixed by heavy metal (Imran et al., 2018). In this study, DCAP was found to have a positive effect on soil P availability (Tables 3 and 6). However, it did not decrease the concentrations of Fe and Al as well as the soil acidity compared with that in the application of an equivalent amount of untreated P. Jeffrey & Hopkins (2013) reported that DCAP coating provided a compound with a high negative-charge density that quickly dissolves in soil solution. Therefore, it helps increase P solubility and creating P more accessible for crop uptake.

AVAIL-coated DCAP fertilizers reduced the over-release of P concentration in soil solution, thus reducing P precipitation by toxic metals such as Al and Fe (Hopkins et al., 2018). According to Imran et al. (2018), the use of DCAP-coated P fertilizer significantly increased the biomass and P accumulation of wheat cultivated in alkaline calcareous soil. In the current study, DCAP coating on P fertilizer improved the P concentration in the stem and tuber of sweet potato as well as dry biomass, resulting in increased total P accumulation (Tables 5 and 7). Similar results were reported by Noor et al. (2017), Imran et al. (2018), and Aziz et al. (2020). They demonstrated that DCAP coating on P fertilizer improved P concentrations in plants and enhanced the total P uptake of crops.

A strong correlation was observed between sweet potato yield and the concentration of available P in soil in both the pot and field experiments (Figs. 1 and 2). The result was line in with the report of Dang, Ngoc & Hung (2021), who indicated that there was a positive correlation between pomelo productivity and available P (*R*^*2*^ = 0.75). P is a macroelement that limits crop growth and yield (Fosu-Mensah & Mensah, 2016). According to Ros et al. (2020), crop yield was significantly increased by increasing soil P availability. Another result also indicated that the content of available P in soil is a key factor significantly affecting plant productivity (Bhat et al., 2017; Johan et al., 2021).

The use of P fertilizer coated with DCAP before application into the soil has reduced the amount of P fertilizer applied to sweet potatoes. This may mitigate surface and groundwater pollution and also reduce the risk of heavy metal (arsenic and cadmium) contamination in agricultural soil (Jiao et al., 2012). According to Noor et al. (2017), another benefit of DCAP is its ability to decrease the amount of P fertilizer for plants, which reduces the cost of production, thus improving economic efficiency.

To sum up, the use of P fertilizer coated with DCAP was beneficial to enhance the P availability and improve the diameter and length of sweet potato tuber, resulting in increased tuber yield (Tables 3 and 4). Furthermore, we found that applying phosphate fertilizer with coated DCAP reduced the amount of chemical P fertilizer use by 25% compared to that with phosphate fertilizer without coated DCAP, but sweet potato yield as well as P available content in the soil were not reduced. (Tables 6 and 7). This result represent a considerable step forward in the pursuit of sustainable agriculture and improving the income of farmers by decreasing the cost of fertilizer. Hence, we recommend the use of P fertilizer coated with DCAP on acidic soil in the VMD.

More research is needed to investigate the effects of DCAP on P-Al and P-Fe concentrations in acidic soils, which are factors that significantly influence the availability of P in the soil. Moreover, the study of K availablity content in ASSs is necessary because available K may be affected by key reasons, including soil texture, mineralogy, temperature, and soil acidity (Rawal et al., 2022). In addition, the relationship between K and P availability in acidic soils need to be clarified in the next studies.

## Conclusions

The results of the pot experiments indicated that the use of DCAP-coated P fertilizer enhanced the concentration of available P (~10%) in soil as well as the tuber yield (~11%–15%) compared with the use of P fertilizer not coated with DCAP. However, soil chemical properties (pH, total P, and exchangeable Al and Fe) were not affected by DCAP. The field trials demonstrated the superior efficacy of DCAP when coated on P fertilizer. The available P content in the two sites (Vinh Long and Dong Thap) treated with DCAP significantly increased by 30%–35%. Therefore, tuber yield and total P uptake in the treatment of 75% P dose recommendation + DCAP was not significantly different with the treatment of 100% P fertilizer, saving 25% P compared with the traditional application utilized in sweet potato fields in the VMD. In short, using DCAP-coated P fertilizer could effectively improve sweet potato growth, yield, and P efficiency compared with that of uncoated P fertilizer.

## Supplemental Information

10.7717/peerj.14803/supp-1 Supplemental Information 1 Raw data.

## Additional Information and Declarations

### Field Study Permissions

The following information was supplied relating to field study approvals (*i.e*., approving body and any reference numbers): Field experiments were approved by Tran Van Bay and Nguyen Van Thuan. They are farmers and own these soils, with the contract number: 101/2019/HĐ-NCKH and 105/2019/HĐ-NCKH, respectively.

### Data Availability

The following information was supplied regarding data availability: The raw data is available in the Supplemental File.
